# Five-year outcomes of percutaneous coronary intervention and coronary artery bypass grafting for multivessel disease: a national population-based study of regional practice

**DOI:** 10.1093/ehjopen/oeag043

**Published:** 2026-03-18

**Authors:** Muhammad Rashid, Florence Lai, Suraj Pathak, Weiqi Liao, Hardeep Aujla, Sarah Murray, Jeremy Dearling, Ann Cheng, Robert Grant, Nick Curzen, Mamas A Mamas, Gavin J Murphy

**Affiliations:** Leicester NIHR Biomedical Research Centre, Leicester BHF Centre for Research Excellence, Department of Cardiovascular Sciences, University of Leicester, Clinical Sciences Wing, Glenfield Hospital, Groby Road, Leicester LE3 9QP, UK; Keele Cardiovascular Research Group, School of Medicine, Keele University, ST5 5BG, UK; Leicester NIHR Biomedical Research Centre, Leicester BHF Centre for Research Excellence, Department of Cardiovascular Sciences, University of Leicester, Clinical Sciences Wing, Glenfield Hospital, Groby Road, Leicester LE3 9QP, UK; Leicester NIHR Biomedical Research Centre, Leicester BHF Centre for Research Excellence, Department of Cardiovascular Sciences, University of Leicester, Clinical Sciences Wing, Glenfield Hospital, Groby Road, Leicester LE3 9QP, UK; Leicester NIHR Biomedical Research Centre, Leicester BHF Centre for Research Excellence, Department of Cardiovascular Sciences, University of Leicester, Clinical Sciences Wing, Glenfield Hospital, Groby Road, Leicester LE3 9QP, UK; Leicester NIHR Biomedical Research Centre, Leicester BHF Centre for Research Excellence, Department of Cardiovascular Sciences, University of Leicester, Clinical Sciences Wing, Glenfield Hospital, Groby Road, Leicester LE3 9QP, UK; Leicester NIHR Biomedical Research Centre, Leicester BHF Centre for Research Excellence, Department of Cardiovascular Sciences, University of Leicester, Clinical Sciences Wing, Glenfield Hospital, Groby Road, Leicester LE3 9QP, UK; National Cardiac Surgery Patient and Public Involvement Group, Department of Cardiovascular Sciences, Glenfield Hospital, Leicester LE3 9QP, UK; National Cardiac Surgery Patient and Public Involvement Group, Department of Cardiovascular Sciences, Glenfield Hospital, Leicester LE3 9QP, UK; Leicester NIHR Biomedical Research Centre, Leicester BHF Centre for Research Excellence, Department of Cardiovascular Sciences, University of Leicester, Clinical Sciences Wing, Glenfield Hospital, Groby Road, Leicester LE3 9QP, UK; Leicester NIHR Biomedical Research Centre, Leicester BHF Centre for Research Excellence, Department of Cardiovascular Sciences, University of Leicester, Clinical Sciences Wing, Glenfield Hospital, Groby Road, Leicester LE3 9QP, UK; Faculty of Medicine, University of Southampton & Department of Cardiology, Southampton University Hospitals NHS Trust Southampton, SO16 6YD UK; Keele Cardiovascular Research Group, School of Medicine, Keele University, ST5 5BG, UK; National Institute for Health and Care Research (NIHR) Birmingham Biomedical Research Centre, Birmingham, B15 2TH, UK; Leicester NIHR Biomedical Research Centre, Leicester BHF Centre for Research Excellence, Department of Cardiovascular Sciences, University of Leicester, Clinical Sciences Wing, Glenfield Hospital, Groby Road, Leicester LE3 9QP, UK

**Keywords:** Myocardial revascularization, Multivessel PCI, CABG, Inequality

## Abstract

**Aims:**

To study how the regional preferences for less invasive multivessel coronary revascularization would adversely affect long-term clinical outcomes.

**Methods:**

A national retrospective cohort study utilizing instrumental variable analysis to estimate the causal effect of revascularization strategy.

England, using national linked Hospital Episode Statistics (HES) with office of national statistic mortality data from 2007 to 2020.

The analysis included 173 771 individuals with complete 5-year follow-up who underwent multivessel revascularization for coronary artery disease. Of this cohort, 63 189 (36.4%) received percutaneous coronary intervention (PCI) and 110 582 (63.6%) received coronary artery bypass grafting (CABG). In total, 37 894 (21.8%) participants were female, and 153 048 (88.2%) were of White ethnicity.

The exposure was the preference between multivessel PCI or CABG. The regional ratio of CABG-to-PCI procedures was used as the instrumental variable.

The primary outcome was all-cause mortality, assessed in-hospital and up to 5 years post-procedure.

**Results:**

The all-cause mortality was 2.1% (*n* & 3587) in-hospital and 16.4% (*n* & 28 474) at 5 years. The proportion of patients undergoing CABG varied significantly across regions (25.4–82.3%), demonstrating validity as an instrumental variable. In the primary analysis, CABG was associated with higher in-hospital all-cause mortality vs. PCI [average treatment effect (ATE), 1.1%; 95% confidence interval (CI), 0.6–1.6%] but lower 5-year all-cause mortality (ATE, −5.4%; 95% CI, −7.0 to −3.7%). Adjusted hazard ratios stratified by quartiles of regional CABG-to-PCI ratios showed an increase in in-hospital mortality but a decrease in 5-year mortality as the proportion of CABG increased.

**Conclusion:**

Regional preferences for revascularization with multivessel PCI result in lower in-hospital all-cause mortality, a key quality metric, but worse long-term outcomes for individuals with multivessel coronary artery disease.

What is already known on this topic Coronary artery bypass grafting (CABG) and percutaneous coronary intervention (PCI) are established treatments for multivessel coronary disease. We searched PubMed/MEDLINE and relevant cardiology guidelines and systematic reviews up to early 2025 using terms including ‘coronary artery bypass’, ‘percutaneous coronary intervention’, ‘multivessel disease’, ‘regional variation’, ‘outcomes’, ‘mortality’, and ‘quality metrics’. Randomized trials, including the 10-year SYNTAX follow-up, show long-term CABG survival benefits over PCI, particularly in complex disease, despite higher initial procedural risks. However, significant regional variation in real-world revascularization practice persists globally, and quality metrics often prioritize short-term (e.g. in-hospital) mortality. The causal impact of these real-world practice variations on long-term population outcomes, accounting for unmeasured confounding, remained unclear.

Added value of this study This large English national cohort study (∼174 000 patients, 2009–2015) is the first to our knowledge to use instrumental variable analysis, leveraging regional practice variation as a natural experiment, to estimate the causal effect of real-world revascularization strategy preference (CABG vs. multivessel PCI) on outcomes. By mitigating measured and unmeasured confounding, we quantified a distinct trade-off: regional practice patterns favouring CABG were associated with higher in-hospital mortality [average treatment effect (ATE), +1.1%; 95% CI, 0.6– 1.6%] but substantially lower 5-year all-cause mortality (ATE, –5.4%; 95% CI, –7.0 to −3.7%) compared to patterns favouring PCI. These results strongly challenge the adequacy of quality metrics focused primarily on in-hospital or other short-term mortality endpoints, suggesting they might inadvertently incentivize practice associated with worse long-term population health. There is an urgent need to incorporate long-term outcomes into quality assessment frameworks, promote standardized care pathways considering durable benefits, and better guide decisions for complex patients often underrepresented in trials.

## Introduction

Myocardial revascularization with coronary artery bypass grafting (CABG) or percutaneous coronary intervention (PCI) remains the cornerstone of managing severe symptomatic multivessel coronary artery disease (CAD)^1,2^ In-hospital mortality is used as a metric for comparing the outcomes of revascularization across institutions and regions, as it captures the immediate impact of procedural complications, such as bleeding, stroke, or myocardial infarction.^3,4^ Randomized controlled trials (RCTs) show that CABG carries a 1–2% higher risk of in-hospital mortality than PCI, primarily due to the invasiveness of the procedure and associated complications, such as sternal wound infections and stroke.^5,6^ However, using in-hospital mortality as a quality metric may apparently favour PCI, even in patients who derive greater long-term benefit from CABG, such as those with complex multivessel disease or diabetes.^5,7,8^

We previously identified unwarranted, regional variation in multivessel revascularization practices across England, with a clear preference towards PCI over CABG in some areas.^9,10^ This variation presents an opportunity for a quasi-experimental study using an instrumental variable approach and causal inference methods that mitigate important sources of bias in observational analyses to compare the effects of regional preference for CABG vs. multivessel PCI on short- and long-term outcomes. The aim of this study was to explore whether regions with greater use of multivessel PCI demonstrate differences in short-term outcomes, such as in-hospital mortality, and whether these patterns translate into differences in long-term outcomes, including mortality and major adverse cardiovascular events (MACE), compared to regions favouring CABG. The results address a gap in our knowledge of the real-world implications of revascularization choices beyond the index hospitalization and highlight the limitations of in-hospital mortality as a quality metric.

## Methods

### Design

This population-based cohort study analysed routinely collected healthcare data from all National Health Service (NHS) hospitalizations in England^11,12^ The study received appropriate governance approvals from the University of Leicester Research Ethics Committee, and data access was approved by NHS Digital (22322-yll15-ls:cardiovascularsciences). The research adhered to the STROBE (Strengthening the Reporting of Observational Studies in Epidemiology) and EHR CODE (Electronic Health Records for Clinical Outcomes and DEsign) guidelines for reporting of observational analyses.^13–15^ This study was planned, conducted, and written in collaboration with representatives from national patient groups, whose contributions are recognized through co-authorship.

### Data sources

Data were sourced from the Hospital Episodes Statistics Admitted Patient Care (HES-APC) dataset from 1 January 2007 to 31 December 2020^11,16^ Hospital Episodes Statistics Admitted Patient Care includes demographic, geographic, diagnostic (ICD-10 coded), and procedural (OPCS-4 coded) data, with a diagnostic coding accuracy of 96% and a procedural coding accuracy of 97%.^11^ Mortality outcomes were ascertained by linkage to the UK National Death Registry (Office for National Statistics, ONS).^17^

### Cohort

The analysis cohort included patients admitted to NHS hospitals in England between 1 April 2009 and 1 April 2015 who underwent revascularization (CABG or PCI) within the subsequent 2 years and had complete 5-year follow-up data.

### Case ascertainment and exposures

Phenotyping algorithms for defining study cohorts, inclusion and exclusion criteria, patient comorbidities, and outcomes of interest are provided in Supplementary material online, *Tables S1*–*S3*. Patient demographics, including age, sex, ethnicity, and index of multiple deprivation (IMD), were extracted from the index episode. Diagnoses and procedures performed in all hospital episodes within 2 years prior to the index episodes were used to establish patients’ medical histories. We defined index episodes as the hospital episodes in which revascularization (CABG or multivessel PCI) was performed. Coronary artery bypass grafting was defined by OPCS-4 codes K40–K46. To identify a cohort where multivessel coronary disease was treated percutaneously and where CABG might be a viable alternative, we included only patients undergoing multivessel PCI in the PCI group. Non-multivessel PCI was excluded as we judged that surgery would be less likely to be considered or indicated in these patients. Similarly, patients undergoing concomitant valve intervention or thoracic aorta surgery were excluded. Multivessel PCI was defined by either (i) a single PCI involving multiple coronary arteries (OPCS4 K492) or deployment of three or more stents (OPCS4 K752, K754) or (ii) a staged PCI where the patient underwent more than one PCI intervention (OPCS-4 K49, K50, K75) within 90 days of their first procedure. This definition aims to capture patients with multivessel CAD treated with PCI and aligns broadly with contemporary trials comparing CABG vs. PCI for multivessel revascularization.^18,19^ To ensure our findings were not biased by planned staged interventions, a sensitivity analysis was performed defining repeat revascularization as any unplanned procedure occurring between 90 days and 5 years post-index.

### Outcomes

Outcomes were measured using diagnoses recorded in hospital episodes following the index episode and linkage to the UK National Death Registry. The primary outcome was all-cause mortality, chosen for its importance to patients, use in contemporary RCTs of myocardial revascularization, and low risk of detection bias. Secondary outcomes included cardiovascular mortality (CVM), acute myocardial infarction (AMI), acute coronary syndrome (ACS), stroke [cerebrovascular accident (CVA)], heart failure hospitalization (HFH), any cardiovascular hospitalization (CVH), and a composite outcome of MACE, encompassing any of the above. Patients were followed for a minimum of 5 years. Hospitalization outcomes were identified using the primary diagnosis recorded in HES, while death outcomes were ascertained from HES and the ONS. To distinguish procedural from non-procedural MI, follow-up for AMI began from the subsequent admission after the index episode. For cardiovascular re-hospitalization, follow-up started the day after the intervention for CABG or the first multivessel PCI. For staged PCI, follow-up began from the first PCI within the 90-day window.

## Statistical analysis

Descriptive statistics and baseline comparisons: Continuous variables were expressed as medians with interquartile ranges (IQRs) and categorical variables as numbers with percentages. Group comparisons were performed using the Mann–Whitney U test for continuous variables and Pearson’s χ^2^ test for categorical variables. Missing ethnicity and IMD data were imputed from prior hospital episodes within a 2-year look-back period. Patients with persistent missing data were excluded from analyses requiring complete covariates. All analyses used two-sided *P*-values, with statistical significance set at *P* < 0.05.

Instrumental variable analysis (IVA): Instrumental variable analysis was used to estimate the effects of regional revascularization preference (CABG vs. multivessel PCI) on primary and secondary outcomes. Instrumental variable analysis is a two-stage regression method that accounts for observed and unobserved confounders. The first-stage regression models the relationship between the instrumental variable and treatment selection, while the second-stage regression models the relationship between the outcome and the predicted probability of treatment selection. As described previously, a Recursive Bivariate Probit (RBiProbit; Stata) model was used.^9,10^ Both stages controlled for prespecified covariates: age, sex, ethnicity, IMD, and comorbidities [diabetes, hypertension, hyperlipidaemia, chronic kidney disease (CKD), previous stroke, and previous MI]. Interactions between mortality and ratio quartiles tested whether short-term outcomes modified long-term effects across regional treatment preferences, using robust standard errors. The instrumental variable model estimated average treatment effects (ATEs) with 95% confidence intervals (CIs), interpreted as absolute risk differences.

The instrumental variable was regional variation in preference for CABG or PCI, derived from patients’ residential outward postcodes at the time of the index procedure. This variable uses geographic variation in practice patterns, which is assumed to influence treatment allocation (conditional on measured covariates) but not be directly related to individual patient prognosis other than through the treatment received, thus mimicking aspects of randomization.^20,21^ A funnel plot (FunnelplotR, R-package) was used to investigate regional variations in CABG rates by plotting standardized CABG ratios against the expected number of CABG surgeries in each region. A logistic model adjusted for age, sex, comorbidities, ethnicity, and IMD estimated the expected number of surgeries. Instrumental variable (IV) strength was assessed using the first-stage F-statistic and Stock-Yogo’s critical values (threshold > 10 indicates a low risk of weak instrument bias).

Subgroup analyses: Treatment effect heterogeneity was explored for in-hospital and 5-year mortality across subgroups: age (>75 vs. ≤ 75 years), sex, ethnicity, deprivation, peripheral vascular disease (PVD), CKD, MI, heart failure (HF), and diabetes. Interaction terms were generated for each subgroup, and RBiProbit models were fitted, adjusting for all covariates and interaction terms. Average treatment effects were calculated for each subgroup.

Secondary analyses: Patients were categorized into quartiles based on the regional CABG-to-PCI ratio for their postcode area. Adjusted survival analyses using Weibull survival models assessed associations between these quartiles and primary and secondary outcomes at 1, 3, and 5 years, adjusting for the same confounders as the IVA model and clustering by region. Propensity score matching (PSM) was performed using nearest-neighbour matching without replacement to compare outcomes across quartiles, with the lowest quartile as the reference. Propensity scores were estimated using logistic regression, adjusting for demographics, comorbidities, and socioeconomic factors. The teffects psmatch command in Stata estimated ATEs for 1-year, 3-year, and 5-year outcomes.

A two-step analysis examined the relationship between in-hospital mortality and long-term survival. First, random-effects logistic regression estimated adjusted in-hospital mortality probabilities by patient postcodes, adjusting for revascularization, age, sex, diabetes, hypertension, hyperlipidaemia, CKD, prior stroke, prior MI, HF, PVD, frailty risk score (HFRS), IMD, ethnicity, and Charlson Comorbidity Index (CCI). Probabilities were aggregated to Clinical Commissioning Group (CCG) level and categorized into quartiles. Second, Weibull regression assessed associations with 1-year, 3-year, and 5-year mortality, incorporating the same covariates and quartiles of adjusted in-hospital mortality and CABG-to-PCI ratios.

Counterfactual 5-year mortality probabilities were estimated using logistic regression with revascularization type, age, sex, diabetes, CKD, HF, PVD, HFRS, IMD, ethnicity, CCI, and year as covariates, clustering standard errors by region. Average predicted probabilities were calculated for two scenarios using the margins command: (i) predicting mortality if all patients in the cohort had hypothetically undergone CABG and (ii) predicting mortality if all had undergone multivessel PCI. The absolute risk reduction (ARR) was calculated as the difference in the mean predicted probabilities. To provide a more conservative estimate reflecting clinical applicability, this ARR was then adjusted by multiplying it by the proportion of screened patients deemed suitable for randomization (i.e. suitable for either PCI or CABG) in the SYNTAX trial^22^ (41.5%, derived from the trial’s CONSORT diagram). The number needed to treat (NNT) with CABG vs. multivessel PCI to prevent one death over 5 years among suitable patients was potentially calculated as 1/adjusted ARR. Adjusted potential lives saved over 5 years at the cohort level were estimated as adjusted ARR × cohort size. Finally, to utilize the full breadth of the available data (extending to 31 December 2020), a sensitivity analysis was performed using multivariable Cox proportional hazards models. In this analysis, follow-up duration was defined from the index procedure to the date of death or administrative censoring at the study end date. The median follow-up for the full cohort was 7.68 years (IQR 5.72–9.84), extending to a maximum of 12.2 years.

All analyses were performed using R (version 4.2) and Stata (version 17). Data management was conducted using PostgreSQL (version 13) and DataGrip (version 2022.2)

## Results

### Study cohort

The study cohort comprised 173 771 patients who underwent revascularization in England between 1 April 2009 and 1 April 2015. Of these, 110 582 (63.6%) underwent CABG, while 63 189 (36.4%) underwent multivessel PCI (see Supplementary material online, *Figure S1*). The median age of the cohort was 68 years (IQR 60–75), with 37 894 (21.8%) being female. Ethnicity distribution included 13 311 (7.7%) patients of Asian ethnicity, 1597 (0.9%) patients of Black ethnicity, and 153 048 (88.2%) patients of White ethnicity. The most socio-economically deprived quintile (20%) included 34 549 patients.

Patients undergoing CABG were older (median age, 69 years) compared to those receiving multivessel PCI (median age, 66 years). The multivessel PCI group had a higher proportion of females (24.1% vs. 20.5%) and patients from less deprived areas. In contrast, the CABG group had a higher burden of comorbidities, including hypertension (76.9% vs. 47.3%), diabetes (29.1% vs. 19.8%), and HF (18.3% vs. 9.0%) (*Table 1*).

**Table 1:** Baseline characteristics of patients undergoing multivessel percutaneous coronary intervention compared to coronary artery bypass grafting

Characteristic	Total (*n* & 173 771)	Multivessel PCI group (*n* = 63 189)	CABG group (*n* = 110 582)
*n*	173 771 (100.0%)	63 189 (36.4%)	110 582 (63.6%)
Age (years), median (IQR)	68(60–75)	66 (57–75)	69 (61–75)
IMD category			
Most deprived	34 596 (20.0%)	12 126 (19.3%)	22 470 (20.5%)
Moderately deprived	34 530 (20.0%)	12 235 (19.4%)	22 295 (20.3%)
Middle level of deprivation	34 619 (20.0%)	12 454 (19.8%)	22 165 (20.2%)
Less deprived	34 495 (20.0%)	12 829 (20.4%)	21 666 (19.7%)
Least deprived	34 549 (20.0%)	13 302 (21.1%)	21 247 (19.3%)
Female	37 894 (21.8%)	15 234 (24.1%)	22 660 (20.5%)
Ethnicity			
White	153 048 (88.2%)	54 957 (87.1%)	98 091 (88.9%)
Asian	13 311 (7.7%)	4845 (7.7%)	8466 (7.7%)
Black	1597 (0.9%)	709 (1.1%)	888 (0.8%)
Mixed	3560 (2.1%)	1290 (2.0%)	2270 (2.1%)
Others	1938 (1.1%)	1294 (2.1%)	644 (0.6%)
Hypertension	114 953 (66.2%)	29 890 (47.3%)	85 063 (76.9%)
Charlson Comorbidity Index			
No comorbidities	16 420 (9.4%)	4306 (6.8%)	12 114 (11.0%)
Mild (one comorbidity)	44 905 (25.8%)	16 771 (26.5%)	28 134 (25.4%)
Moderate (two comorbidities)	34 430 (19.8%)	13 556 (21.5%)	20 874 (18.9%)
Severe (three or more)	78 016 (44.9%)	28 556 (45.2%)	49 460 (44.7%)
Frailty category			
Fit	86 896 (50.0%)	34 503 (54.6%)	52 393 (47.4%)
Mild	29 767 (17.1%)	9967 (15.8%)	19 800 (17.9%)
Moderate	17 738 (10.2%)	5793 (9.2%)	11 945 (10.8%)
Severe	39 370 (22.7%)	12 926 (20.5%)	26 444 (23.9%)
Peripheral vascular disease (PVD)	12 219 (7.0%)	2761 (4.4%)	9458 (8.6%)
Heart failure	25 945 (14.9%)	5709 (9.0%)	20 236 (18.3%)
Acute myocardial infarction (AMI) admission	55 575 (32.0%)	20 309 (32.1%)	35 266 (31.9%)
Cerebrovascular accident (CVA)	8288 (4.8%)	1401 (2.2%)	6887 (6.2%)
Chronic kidney disease	16 266 (9.4%)	4359 (6.9%)	11 907 (10.8%)
Lipidaemia	97 429 (56.1%)	21 319 (33.7%)	76 110 (68.8%)
Diabetes	44 717 (25.7%)	12 493 (19.8%)	32 224 (29.1%)

Unadjusted in-hospital mortality was higher in the CABG group (2.2% vs. 1.7%). However, at 5 years, the CABG group demonstrated lower rates of all-cause mortality (15.8% vs. 17.6%), CVM (12.5% vs. 14.0%), and MACE (42.4% vs. 54.7%) compared to the multivessel PCI group (*Table 2*)

**Table 2:** Crude outcomes stratified according to multivessel percutaneous coronary intervention vs. coronary artery bypass grafting group

Time	Outcome	Total (*n* = 173 771)	Multivessel PCI group (*n* = 63 189)	CABG group (*n* = 110 582)
In-hospital	In-hospital mortality	3587 (2.1%)	1104 (1.7%)	2483 (2.2%)
1 year	All-cause mortality	9539 (5.5%)	3637 (5.8%)	5902 (5.3%)
	Cardiovascular mortality	8742 (5.0%)	3294 (5.2%)	5448 (4.9%)
	AMI hospitalization	3497 (2.0%)	2085 (3.3%)	1412 (1.3%)
	ACS hospitalization	6723 (3.9%)	4160 (6.6%)	2563 (2.3%)
	Heart failure hospitalization	5109 (2.9%)	1710 (2.7%)	3399 (3.1%)
	Any stroke hospitalization	1424 (0.8%)	471 (0.7%)	953 (0.9%)
	Repeat revascularization > 90days	3486 (2.0%)	2248 (3.6%)	1238 (1.1%)
	Any cardiovascular hospitalization	36 261 (20.9%)	16 920 (26.8%)	19 341 (17.5%)
	Major adverse cardiovascular events	43 290 (25.0%)	19 474 (30.9%)	23 816 (21.6%)
3 years	All-cause mortality	18 177 (10.5%)	7252 (11.5%)	10 925 (9.9%)
	Cardiovascular mortality	15 157 (8.7%)	6048 (9.6%)	9109 (8.3%)
	AMI hospitalization	6652 (3.8%)	4071 (6.5%)	2581 (2.3%)
	ACS hospitalization	11 910 (6.9%)	7317 (11.6%)	4593 (4.2%)
	Heart failure hospitalisation	8224 (4.7%)	2979 (4.7%)	5245 (4.8%)
	Any stroke hospitalization	3907 (2.3%)	1332 (2.1%)	2575 (2.3%)
	Repeat revascularization > 90 days	7123 (4.1%)	4411 (7.0%)	2712 (2.5%)
	Any cardiovascular hospitalization	54 236 (31.3%)	24 762 (39.2%)	29 474 (26.7%)
	Major adverse cardiovascular events	65 111 (37.5%)	28 746 (45.6%)	36 365 (33.0%)
5 years	All-cause mortality	28 474 (16.4%)	11 089 (17.6%)	17 385 (15.8%)
	Cardiovascular mortality	22 605 (13.0%)	8850 (14.0%)	13 755 (12.5%)
	AMI hospitalization	9739 (5.6%)	5721 (9.1%)	4018 (3.6%)
	ACS hospitalization	15 794 (9.1%)	9347 (14.8%)	6447 (5.8%)
	Heart failure hospitalization	11 321 (6.5%)	4001 (6.3%)	7320 (6.6%)
	Any stroke hospitalization	6366 (3.7%)	2140 (3.4%)	4226 (3.8%)
	Repeat revascularization > 90 days	9786 (5.7%)	5749 (9.1%)	4037 (3.7%)
	Any cardiovascular hospitalization	66 505 (38.3%)	29 256 (46.4%)	37 249 (33.8%)
	Major adverse cardiovascular events	81 321 (46.9%)	34 539 (54.7%)	46 782 (42.4%)

### Regional variation in coronary artery bypass grafting-to-percutaneous coronary intervention preference

**Figure 1:**
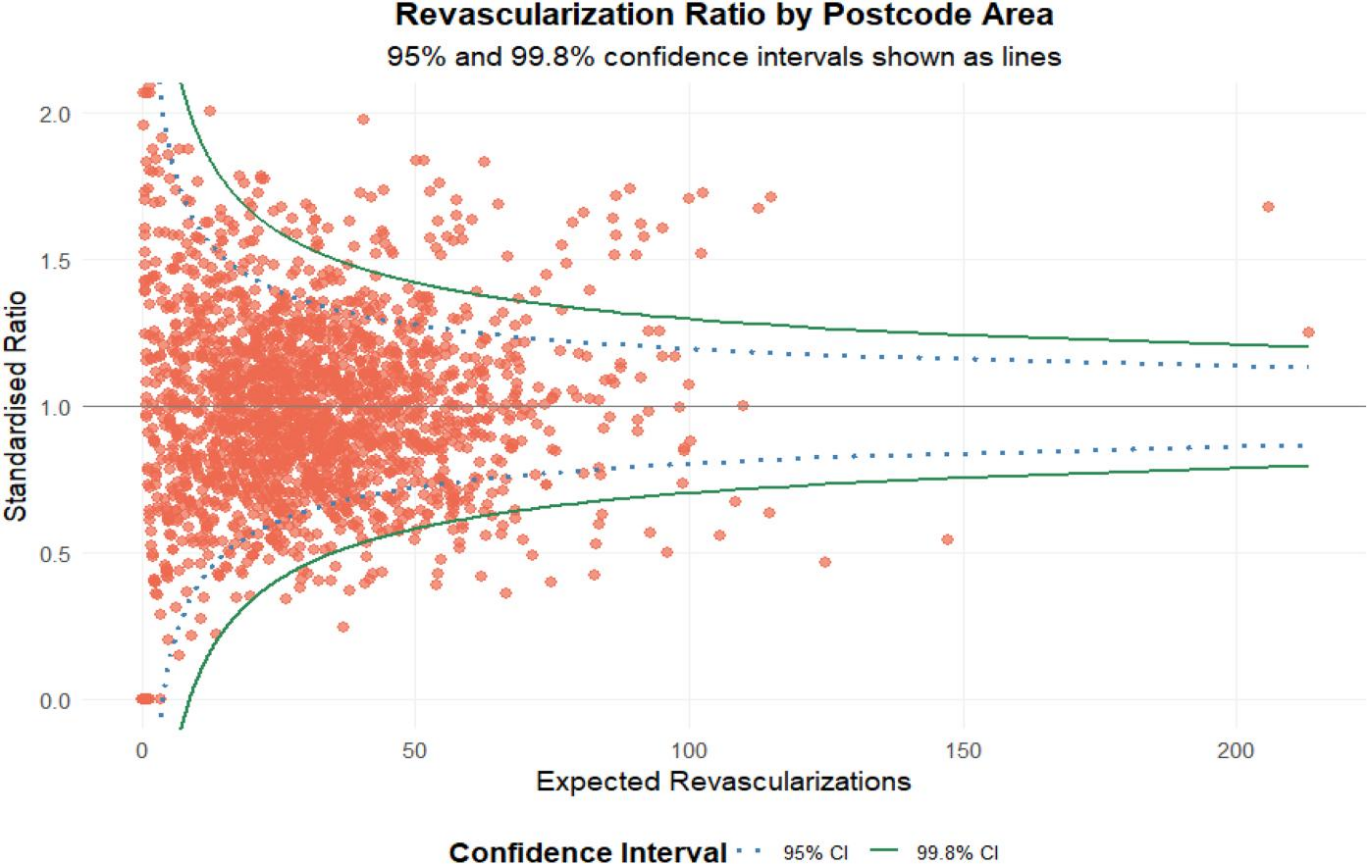
Funnel plot demonstrating the revascularization rate by outward postcode.

Coronary artery bypass grafting rates varied significantly across postcodes in England, ranging from 25.4 to 82.3% (mean, 63.6%; SD, 10.1%; Supplementary material online, *Figure S2*). A funnel plot revealed significant variation after adjusting for known confounders, with 126 (6.2%) regions falling outside the 99.8% CI and 358 (17.6%) outside the 95% CI (*Figure 1*). Regional practice patterns remained highly stable throughout the study period; the correlation between regional CABG-to-PCI ratios in 2009 and 2015 was *r* = 0.82 (*P* < 0.001), and visual inspection of annual ratios confirmed a persistent geographic variation in treatment strategy (see Supplementary material online, *Figure S3*).

### Primary analysis

In the IVA, the CABG-to-PCI ratio demonstrated a strong first-stage F-statistic of 7440.35 (*P* < 0.001), exceeding Stock–Yogo’s threshold of 16.38, confirming its validity as an instrumental variable. Baseline characteristics were well balanced across quartiles of the CABG-to-PCI ratio, supporting the robustness of the analysis (see Supplementary material online, *Table S4*). The IVA-adjusted treatment effects showed that CABG was associated with higher in-hospital all-cause mortality compared to multivessel PCI, with an ATE of 1.1% (95% CI, 0.6–1.6%)

**Figure 2:**
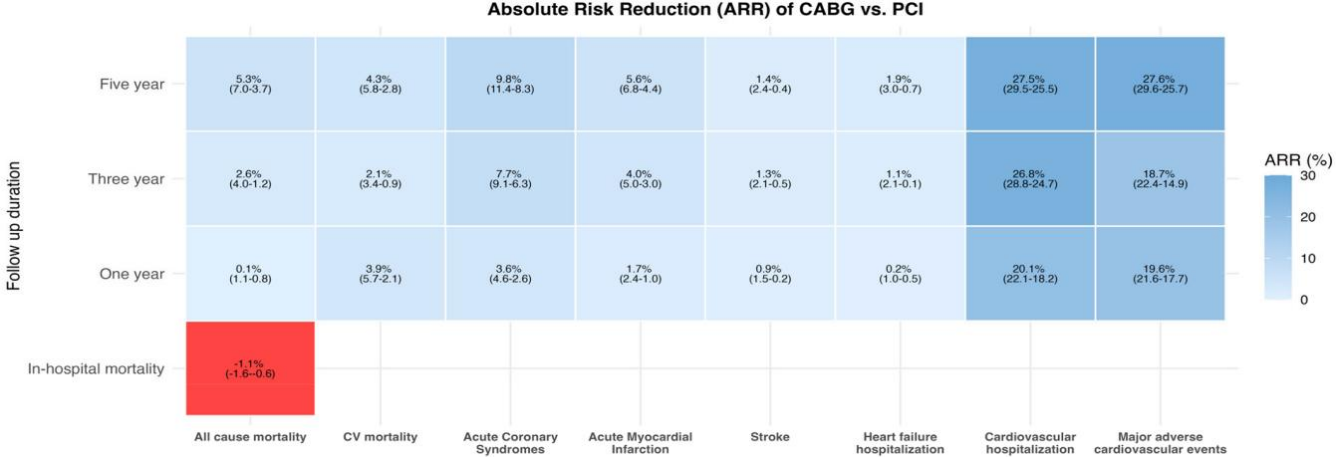
Heatmap illustrating IVA-assessed all-cause mortality and secondary outcomes, comparing multivessel PCI and CABG over time. CABG, coronary artery bypass graft; PCI, percutaneous coronary intervention; IVA, instrumental variable analysis.

At 5 years, CABG was associated with lower all-cause mortality compared to PCI, with an ATE of −5.4% (95% CI, −7.0 to −3.7%). Coronary artery bypass grafting was also associated with lower rates of secondary outcomes at 5 years, including AMI (ATE −5.6%; 95% CI, −6.8 to −4.4%), stroke (ATE −1.4%; 95% CI, −2.4 to −0.4%), HFH (ATE −1.9%; 95% CI, −3.0 to −0.7%), cardiovascular death (ATE −4.3%; 95% CI, −5.8 to −2.8%), and MACE (ATE −27.8%; 95% CI, −30.0 to −25.7%; *Figure 2*; Supplementary material online, *Table S5*). In a sensitivity analysis using standard multivariable Cox proportional hazards models on the full cohort, the survival benefit of CABG remained consistent throughout the follow-up period [adjusted hazard ratio (aHR), 0.80; 95% CI 0.78–0.82; *P* < 0.001], with similar protective effects observed across all secondary endpoints (see Supplementary material online, *Figure S4*).

Analysis at intermediate time points indicated that CABG was associated with significant clinical benefits by 3 years, when ATE for all-cause mortality was −2.6% (95% CI, −4.0 to −1.2%), and for MACE, was −18.7% (95% CI, −22.4 to −14.9%). The benefits of CABG over PCI were consistent across predefined subgroups, including females, people of non-White ethnicity, and those from the most socio-economically deprived regions (see Supplementary material online, *Figure S5*). We performed a sensitivity analysis excluding unplanned repeat revascularizations performed within the first 90 days to avoid including planned staged interventions. Coronary artery bypass grafting was associated with a significant and sustained reduction in late unplanned revascularization at 1 year (ATE, −9.5%; 95% CI, −11.7 to −7.3%), 3 years (ATE, −15.1%; 95% CI, −18.8 to −11.5%), and 5 years (ATE, −16.9%; 95% CI, −20.6 to −13.2%; *P* < 0.001 for all).

### Secondary analyses

**Figure 3:**
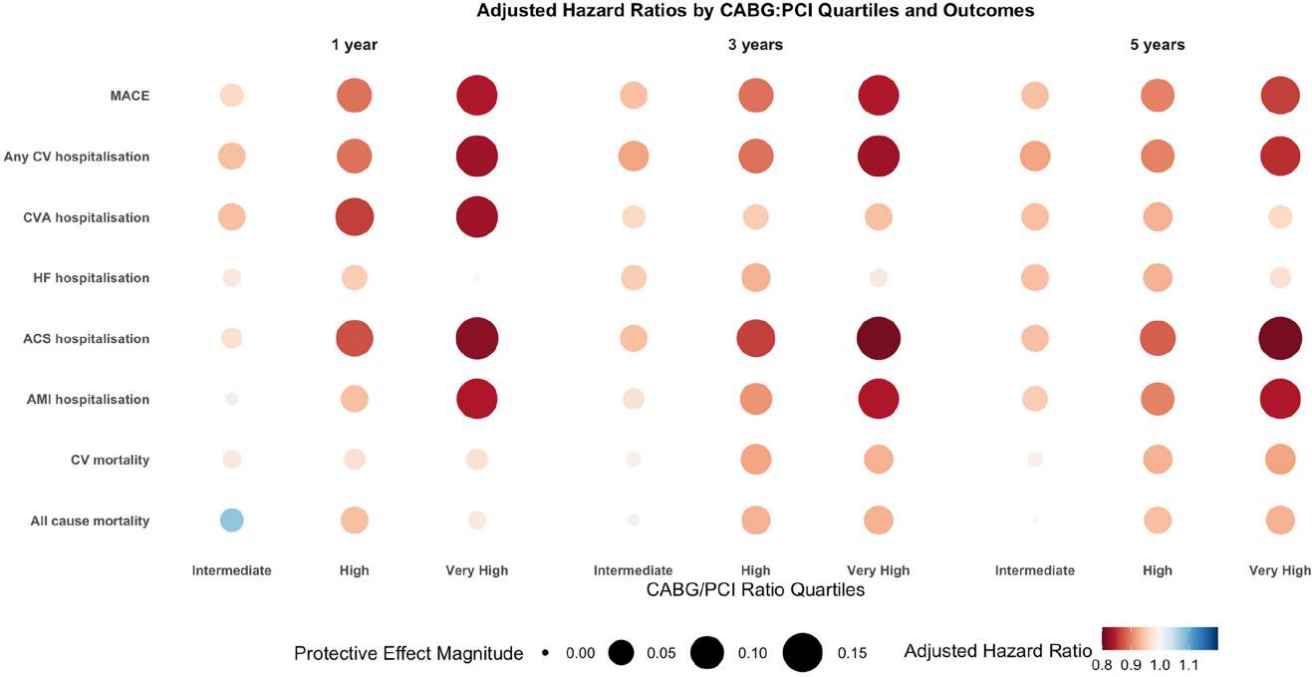
Circle heatmap of adjusted hazard ratio for regional impact of CABG-to-PCI ratio on clinical outcomes at 1, 3, and 5 years. CABG, coronary artery bypass graft; PCI, percutaneous coronary intervention; CV, cardiovascular; HF, heart failure; ACS, acute coronary syndrome; AMI, acute myocardial infarction.

Cox models adjusted for patient factors and regional clustering demonstrated a dose–response relationship, with regions favouring CABG showing progressively better primary and secondary outcomes. Compared to the low CABG-to-PCI ratio quartile, the hazard ratios (HRs) were 0.93 (95% CI, 0.90–0.96) for the very high quartile, 0.94 (95% CI, 0.91–0.97) for the high quartile, and 1.00 (95% CI, 0.97–1.03) for the intermediate quartile (*Figure 3*; Supplementary material online, *Table S6*).

Sensitivity analyses using PSM yielded results consistent with the primary Cox model analysis. The very high CABG-to-PCI quartile was associated with lower all-cause mortality at 3 years (ATE, −0.013; 95% CI, −0.017 to −0.009) and 5 years (ATE, −0.001; 95% CI, −0.004–0.002) (see Supplementary material online, *Table S7*). The PSM results aligned closely with the Cox model, particularly for the very high quartile, where both analyses showed significant reductions in mortality.

In an exploratory analysis, regions with higher in-hospital mortality quartiles were associated with lower 5-year mortality compared to the lowest quartile. The aHRs were 0.88 (95% CI, 0.86–0.91) for the very high quartile, 0.94 (95% CI, 0.91–0.97) for the high quartile, and 0.96 (95% CI, 0.93–0.99) for the intermediate quartile (*Table 3*).

**Table 3:** All-cause mortality outcomes by risk-adjusted mortality quartiles

Follow-up	Risk-adjusted mortality quartile	Adjusted hazard ratio (aHR, 95% CI)
1 year	Low	Ref
1 year	Intermediate	0.97 (0.92–1.03)
1 year	High	0.92 (0.87–0.97)
1 year	Very high	0.80 (0.76–0.85)
3 years	Low	Ref
3 years	Intermediate	0.96 (0.92–1.00)
3 years	High	0.91 (0.87–0.95)
3 years	Very high	0.85 (0.82–0.89)
5 years	Low	Ref
5 years	Intermediate	0.96 (0.93–0.99)
5 years	High	0.94 (0.91–0.97)
5 years	Very high	0.88 (0.86–0.91)

The counterfactual model predicted consistently lower 5-year mortality across all regions if patients hypothetically received CABG compared to multivessel PCI (see Supplementary material online, *Figure S6*). The model estimated a mean 5-year mortality probability of 19.2% under the multivessel PCI scenario vs. 15.0% under the CABG scenario. This represented an initial model-based ARR of 4.1% and a corresponding NNT of 24 (SD 1.3), with a potential number of lives saved of 7157 (SD 395) if all patients received CABG (*Table 4*). However, assuming that the true proportion of all CABG and multivessel PCI patients eligible for both procedures was 41.5%, as reported in the SYNTAX trial, the adjusted ARR was estimated at 1.7%. This corresponds to an adjusted NNT of 58 and suggests that universal adoption of CABG instead of multivessel PCI for this suitable subgroup could have potentially prevented ∼2970 deaths over 5 years.

**Table 4:** Numbers needed to treat and potential lives saved if everyone had coronary artery bypass grafting

Variable	*n*	Mean	SD	25th percentile	Median (50th percentile)	75th percentile
Potential lives saved	173 454	7156.60	395.2	6899.3	7108.7	7390.2
Numbers needed to treat	173 454	24.3	1.3	23.4	24.4	25.1
Adjusted estimate (accounting for 41.5% trial suitability)*:						
Potential lives saved	173 454	2970	134	2712	2911	3228
Numbers needed to treat	173 454	58.4	2.6	53.3	58.3	63.5

* Based on SYNTAX trial.

## Discussion

This study identifies significant regional variation in the use of CABG vs. PCI for multivessel revascularization in England from 2009 to 2015, which persists after adjusting for measured patient characteristics. Using this regional variation as an instrumental variable to mitigate both measured and unmeasured confounding, we found that residing in a region with a higher preference for CABG was associated with higher in-hospital mortality, but significantly lower 5-year all-cause mortality and MACE compared to residing in a region preferring multivessel PCI. These findings were consistent across secondary and sensitivity analyses and key subgroups, including females, the elderly, and non-White individuals. Our exploratory analysis showed that regions with the lowest adjusted in-hospital mortality had the highest 5-year all-cause mortality, suggesting a potential trade-off between short-term procedural safety and long-term outcomes. Our counterfactual modelling suggests this practice variation may have contributed to potentially avoidable deaths during the study period.

### Comparison with existing literature and interpretation of findings

The observed higher in-hospital mortality with CABG is consistent with the results of RCTs.^7,8,18,19,22^ However, the magnitude of the 5-year mortality benefit associated with regional CABG preference in our IVA is considerably larger than the pooled estimates from meta-analyses of RCTs comparing CABG vs. PCI in multivessel and left main disease.^5^ Several factors might explain why our real-world estimate appears larger at 5 years than in RCTs. Firstly, our study reflects real-world practice across all types of hospitals and operators, encompassing a broader and potentially higher-risk patient population than typically enrolled in RCTs, including urgent/emergency cases and patients with comorbidities often leading to trial exclusion. One interpretation is that the long-term benefits of CABG may be greater in high-risk groups typically excluded from trials, although we did not demonstrate this in our subgroup analysis. A significant driver of the long-term benefit was the marked reduction in unplanned late revascularization. Even after excluding the first 90 days to account for staged procedures, CABG was associated with a substantial reduction in late re-intervention, underscoring the superior durability of surgical revascularization in a multivessel CAD cohort. A second interpretation is that these results may reflect residual confounding in the analysis that would favour CABG as these patients are typically fitter at baseline, a crucial determinant of longer-term outcome.

### Drivers of regional variation

The observed regional variation in revascularization preference persists after adjusting for measured patient characteristics, suggesting that local clinical cultures and institutional practices maybe significant drivers of care^23,24^ Furthermore, the regional preference for CABG in this study does not align with known patterns of cardiovascular health inequities^25,26^ These variations often arise in the multidisciplinary Heart Team where decisions are influenced by clinical uncertainty, particularly for complex patients with multiple comorbidities who fall outside the strict inclusion criteria of pivotal RCTs^27,28^ In such scenarios, PCI offers a less invasive option with lower procedural mortality, which may be favoured when surgical risk is perceived as high.^1,2^

Institutional incentives, such as the increasing international trend towards public reporting of risk-adjusted in-hospital mortality, may also play a role.^25,26,29^ While intended to improve quality, such metrics can inadvertently influence treatment decisions towards less invasive options perceived as safer in the short term, potentially at the expense of long-term benefit.^6,22,30^ Our findings of an inverse association between regional in-hospital and 5-year mortality support the hypothesis that a focus on short-term metrics might have unintended long-term consequences.

The use of geographical region as an IV specifically leverages these institutional practice patterns to estimate causal effects. While this approach effectively mitigates individual-level unmeasured confounding, it assumes that regional preference influences outcomes primarily through the treatment received. Although we adjusted for individual-level deprivation and health status, the IV approach acknowledges that regional-level factors are inherent to the practice patterns being studied. This framework provides a ‘real-world’ assessment of how varied clinical strategies across a national healthcare system impact long-term patient survival.

### Implications for clinical practice and policy

This study identifies significant variation in revascularization practices and associated long-term outcomes across England. Regions achieving lower in-hospital mortality through a preference for multivessel PCI appear to do so at the cost of higher 5-year mortality. These findings suggest the value of (i) better evidence (including from real-world data and potentially registries) to guide revascularization decisions, particularly in patients with multiple comorbid conditions who are underrepresented in RCTs, (ii) efforts to standardize care pathways and Heart Team decision-making, potentially through enhanced guideline implementation support and audit, and (iii) development and adoption of quality indicators that encompass meaningful long-term outcomes (like 5-year and potentially 10-year survival), to complement existing measures of procedural safety.^1,2,31^ The long-term data from the SYNTAX trial^22^ underscore the importance of surgical durability in extensive disease. However, the magnitude of the causal effect for MACE observed in our model (ATE −27.8%) likely represents an upper-bound estimate specifically for ‘complex’ patients whose treatment selection was most heavily influenced by regional clinical culture. While this confirms a strong directional benefit for CABG, the absolute magnitude reflects a broad composite endpoint, including repeat hospitalizations, and may not be universally generalizable. Finally, while the 1.1% (95% CI, 0.6–1.6%) absolute increase in in-hospital mortality associated with CABG carries systemic weight, representing nearly 1900 additional periprocedural deaths across the cohort. Yet, our counterfactual modelling highlights a potential trade-off; even when conservatively restricted to patients eligible for either modality, the estimated NNT with CABG to prevent one death at 5 years was 58. This suggests that a focus on marginal gains in short-term procedural safety may inadvertently occur at the expense of a substantial long-term survival benefit. These data do not mandate a universal policy change but rather advocate for a balanced clinical perspective that prioritizes long-term patient longevity over periprocedural metrics alone.

### Strengths and limitations

This study has several strengths: its large, nationally representative dataset, including all multivessel coronary revascularization procedures in NHS hospitals in England over the study period, enhances generalizability within this healthcare system; the use of IVA helps mitigate bias from unmeasured confounders inherent in observational studies; and the consistency across multiple analytical approaches strengthens confidence in the findings. However, several limitations must be acknowledged. First, while HES-APC is a robust national resource, it relies on administrative coding (ICD-10 and OPCS-4), which lacks granular anatomical descriptors, such as the precise number of diseased vessels or the presence of left main stem (LMS) involvement. We addressed this by using procedural proxies to define a high complexity multivessel cohort, but we cannot entirely rule out differences in anatomical complexity between the groups. Second, there remains a likelihood of residual confounding despite the IVA approach, particularly regarding clinical variables not captured in administrative data, such as SYNTAX score, ejection fraction, specific reasons for treatment choice, and the formal completeness of revascularization. Third, our definition of repeat revascularization required refinement to distinguish between planned staged interventions and clinical failure; although our sensitivity analysis excluding the first 90 days confirmed the surgical durability of CABG, the potential for individual-level misclassification remains.^10^ Finally, while our study provides mature long-term data, it does not reflect the most recent iterations of PCI technology (e.g. latest-generation drug-eluting stents) or contemporary medical therapy guidelines established after 2015. However, the fundamental biological and mechanical differences between surgical bypass and percutaneous stenting remain consistent, and our study captures the critical long-term window where these differences manifest most clearly in clinical outcomes. The findings may not be fully generalizable to other healthcare systems or more contemporary practice, given ongoing evolution in PCI technology and techniques.

## Conclusions

Analyses of revascularization preferences in a large national dataset reveal significant regional variation in practice associated with divergent short- and long-term outcomes. Regions with lower CABG: multivessel PCI ratios achieved lower in-hospital mortality but experienced higher 5-year mortality and MACE rates. While CABG carries higher upfront risk, our findings suggest that it is associated with superior long-term survival in the context of multivessel disease treated in routine practice. This study highlights an urgent need for better evidence synthesis integrating RCT and real-world data to inform clinical decisions, strategies to promote standardized evidence-based care pathways, and a shift towards quality metrics that prioritize long-term patient outcomes over sole reliance on short-term procedural safety.

## Supplementary Material

oeag043_Supplementary_Data

## Data Availability

The data underlying this article were provided by NHS Digital under licence. The data that support the findings of this study are available from the corresponding author upon reasonable request. Raw data may only be shared with permission from NHS Digital.
