# Supplementary material for: Five-year outcomes of percutaneous coronary intervention and coronary artery bypass grafting for multivessel disease: a national population-based study of regional practice
Source: Eur Heart J Open. 2026 Mar 18;6(2):oeag043. doi: 10.1093/ehjopen/oeag043 (PMC13089547; doi:10.1093/ehjopen/oeag043)

SUPPLEMENTARY MATERIAL

Supplementary Table1: ICD-10 and OPCS-4 Codes for definition of CABG and multivessel PCI

| Category | Code | Description | Additional Notes |
| --- | --- | --- | --- |
| CABG (OPCS-4) | K40X | Saphenous vein graft replacement of coronary artery | Any code between 01/04/2009-01/04/2015 |
| CABG (OPCS-4) | K41X | Other autograft replacement of coronary artery |  |
| CABG (OPCS-4) | K42X | Allograft replacement of coronary artery |  |
| CABG (OPCS-4) | K43X | Prosthetic replacement of coronary artery |  |
| CABG (OPCS-4) | K44X | Other replacement of coronary artery |  |
| CABG (OPCS-4) | K45X | Connection of thoracic artery to coronary artery |  |
| CABG (OPCS-4) | K46X | Other bypass of coronary artery |  |
| High Risk PCI (OPCS-4) | K49X | Transluminal balloon angioplasty of coronary artery | More than one code within 90 days between 01/04/2009-01/04/2015 |
| High Risk PCI (OPCS-4) | K50X | Other therapeutic transluminal operations on coronary artery |  |
| High Risk PCI (OPCS-4) | K75X | Percutaneous transluminal balloon angioplasty and insertion of stent into coronary artery |  |

Supplementary Table 2: ICD-10 Codes for Comorbidities

| Category | Code | Description |
| --- | --- | --- |
| Congestive Heart Failure (CHF) | I50.0 | Congestive heart failure |
| Diabetes Mellitus | E10 | Type 1 diabetes mellitus |
|  | E11 | Type 2 diabetes mellitus |
|  | E12 | Malnutrition-related diabetes mellitus |
|  | E13 | Other specified diabetes mellitus |
|  | E14 | Unspecified diabetes mellitus |
| Chronic Kidney Disease (CKD) | N18 | Chronic kidney disease |
|  | N19 | Unspecified renal failure |
|  | N990 | Post procedural renal failure |
|  | I12X | Hypertensive renal disease |
|  | I13X | Hypertensive heart and renal disease |
| Transient Ischemic Attack/Stroke (TIA/Stroke) | G45 | Transient cerebral ischaemic attacks and related syndromes |
|  | I60 | Subarachnoid haemorrhage |
|  | I61 | Intracerebral haemorrhage |
|  | I62 | Other nontraumatic intracranial haemorrhage |
|  | I63 | Cerebral infarction |
|  | I64 | Stroke, not specified as haemorrhage or infarction |
|  | I65 | Occlusion and stenosis of precerebral arteries, not resulting in cerebral infarction |
|  | I69 | Sequelae of cerebrovascular disease |
| Peripheral Vascular Disease (PVD) | I739 | Peripheral vascular disease |
|  | I74X | Arterial embolism and thrombosis |
| Hypertension | I10 | Essential (primary) hypertension |
|  | I11 | Hypertensive heart disease |
|  | I13 | Hypertensive heart and renal disease |

Supplementary Table 3: ICD-10 Codes for Outcomes

| Category | Code | Description |
| --- | --- | --- |
| Cardiovascular Mortality | I | Diseases of the circulatory system |
|  | I | Diseases of the circulatory system |
|  | I60 | Subarachnoid haemorrhage |
|  | I61 | Intracerebral haemorrhage |
|  | I62 | Other nontraumatic intracranial haemorrhage |
|  | I63 | Cerebral infarction |
|  | I64 | Stroke, not specified as haemorrhage or infarction |
|  | I65 | Occlusion and stenosis of precerebral arteries, not resulting in cerebral infarction |
| Stroke Hospitalization | I60 | Subarachnoid haemorrhage |
|  | I61 | Intracerebral haemorrhage |
|  | I62 | Other nontraumatic intracranial haemorrhage |
|  | I63 | Cerebral infarction |
|  | I64 | Stroke, not specified as haemorrhage or infarction |
|  | I65 | Occlusion and stenosis of precerebral arteries, not resulting in cerebral infarction |
|  | I69 | Sequelae of cerebrovascular disease |
| Myocardial Infarction (MI) hospitalisation | I21 | Acute myocardial infarction |
| Acute Coronary Syndrome (ACS) hospitalisation | I20 | Angina pectoris |
|  | I21 | Acute myocardial infarction |
|  | I22 | Subsequent myocardial infarction |
|  | I23 | Certain current complications following acute myocardial infarction |
|  | I24 | Other acute ischaemic heart diseases |
| Heart Failure hospitalisation | I11 | Hypertensive heart disease with heart failure |
|  | I13.0 | Hypertensive heart and renal disease with heart failure |
|  | I13.2 | Hypertensive heart and chronic kidney disease with heart failure and stage 5 CKD or ESRD |
|  | I50 | Heart failure |
| Stroke Hospitalisation | I60 | Subarachnoid haemorrhage |
|  | I61 | Intracerebral haemorrhage |
|  | I62 | Other nontraumatic intracranial haemorrhage |
|  | I63 | Cerebral infarction |
|  | I64 | Stroke, not specified as haemorrhage or infarction |
|  | I65 | Occlusion and stenosis of precerebral arteries, not resulting in cerebral infarction |
|  | I66 | Occlusion and stenosis of cerebral arteries, not resulting in cerebral infarction |
|  | I69 | Sequelae of cerebrovascular disease |
|  | I60 | Subarachnoid haemorrhage |
|  | I61 | Intracerebral haemorrhage |
|  | I62 | Other nontraumatic intracranial haemorrhage |
|  | I63 | Cerebral infarction |

Supplementary Table 4: Characteristics of Patients Stratified According to CABG:PCI Ratio Quartiles

| Characteristic | Quartile 1 (N=44,438) | Quartile 2 (N=43,630) | Quartile 3 (N=44,786) | Quartile 4 (N=40,600) |
| --- | --- | --- | --- | --- |
| Total | 44,438 (25.6%) | 43,630 (25.2%) | 44,786 (25.8%) | 40,600 (23.4%) |
| Age, mean (SD) | 66.94 (10.95) | 66.56 (10.90) | 66.86 (10.66) | 67.15 (10.44) |
| Female | 9,928 (22.3%) | 9,576 (21.9%) | 9,409 (21.0%) | 8,926 (22.0%) |
| IMD score, mean (SD) | 21.41 (15.21) | 22.33 (15.72) | 19.91 (13.61) | 22.51 (16.54) |
| Ethnicity - White | 38,372 (86.3%) | 36,014 (82.5%) | 39,734 (88.7%) | 38,928 (95.9%) |
| Ethnicity - Asian | 3,784 (8.5%) | 5,079 (11.6%) | 3,626 (8.1%) | 822 (2.0%) |
| Ethnicity - Black | 648 (1.5%) | 529 (1.2%) | 304 (0.7%) | 116 (0.3%) |
| Ethnicity - Mixed | 871 (2.0%) | 1,423 (3.3%) | 766 (1.7%) | 500 (1.2%) |
| Ethnicity - Others | 763 (1.7%) | 585 (1.3%) | 356 (0.8%) | 234 (0.6%) |
| CCI score, mean (SD) | 5.96 (6.36) | 6.08 (6.49) | 5.87 (6.21) | 5.99 (6.29) |
| HFRS, mean (SD) | 9.43 (12.10) | 10.17 (12.70) | 9.45 (12.02) | 9.72 (11.89) |
| Peripheral Vascular Disease (PVD) | 2,893 (6.5%) | 3,035 (7.0%) | 2,996 (6.7%) | 3,216 (7.9%) |
| Heart failure | 5,937 (13.4%) | 6,766 (15.5%) | 7,119 (15.9%) | 6,077 (15.0%) |
| Acute Myocardial Infarction (AMI) | 13,254 (29.8%) | 13,575 (31.1%) | 14,128 (31.5%) | 14,556 (35.9%) |
| Stroke | 1,895 (4.3%) | 2,141 (4.9%) | 2,338 (5.2%) | 1,914 (4.7%) |
| Chronic Kidney Disease (CKD) | 4,200 (9.5%) | 3,975 (9.1%) | 4,365 (9.7%) | 3,714 (9.1%) |
| Lipidaemia | 23,227 (52.3%) | 25,572 (58.6%) | 25,600 (57.2%) | 22,762 (56.1%) |
| Hypertension | 28,091 (63.2%) | 29,591 (67.8%) | 30,136 (67.3%) | 26,886 (66.2%) |
| Diabetes Mellitus | 11,365 (25.6%) | 11,961 (27.4%) | 11,716 (26.2%) | 9,626 (23.7%) |
| **In-hospital mortality** | **894 (2.0%)** | **933 (2.1%)** | **896 (2.0%)** | **864 (2.1%)** |
| 1-year outcomes |  |  |  |  |
| All cause mortality | 2,510 (5.6%) | 2,500 (5.7%) | 2,343 (5.2%) | 2,186 (5.4%) |
| CV mortality | 2,305 (5.2%) | 2,279 (5.2%) | 2,146 (4.8%) | 2,012 (5.0%) |
| Any AMI hospitalisation | 956 (2.2%) | 944 (2.2%) | 859 (1.9%) | 738 (1.8%) |
| Heart failure hospitalisation (HFH) | 1,308 (2.9%) | 1,323 (3.0%) | 1,284 (2.9%) | 1,194 (2.9%) |
| Any ACS hospitalisation | 1,868 (4.2%) | 1,822 (4.2%) | 1,615 (3.6%) | 1,418 (3.5%) |
| **Any CV hospitalisation** | **10,065 (22.6%)** | **9,324 (21.4%)** | **9,032 (20.2%)** | **7,840 (19.3%)** |
| MACE | 11,914 (26.8%) | 11,181 (25.6%) | 10,767 (24.0%) | 9,428 (23.2%) |
| 3-year outcomes |  |  |  |  |
| All cause mortality | 4,867 (11.0%) | 4,636 (10.6%) | 4,524 (10.1%) | 4,150 (10.2%) |
| CV mortality | 4,083 (9.2%) | 3,828 (8.8%) | 3,786 (8.5%) | 3,460 (8.5%) |
| Any AMI hospitalisation | 1,850 (4.2%) | 1,759 (4.0%) | 1,634 (3.6%) | 1,409 (3.5%) |
| Any stroke hospitalisation | 1,006 (2.3%) | 987 (2.3%) | 1,014 (2.3%) | 900 (2.2%) |
| Any ACS hospitalisation | 3,357 (7.6%) | 3,176 (7.3%) | 2,866 (6.4%) | 2,511 (6.2%) |
| **Any heart failure hospitalisation (HFH)** | **2,141 (4.8%)** | **2,109 (4.8%)** | **2,066 (4.6%)** | **1,908 (4.7%)** |
| Any CV hospitalisation | 14,951 (33.6%) | 13,797 (31.6%) | 13,636 (30.4%) | 11,852 (29.2%) |
| MACE | 17,841 (40.1%) | 16,592 (38.0%) | 16,338 (36.5%) | 14,340 (35.3%) |
| 5-year outcomes |  |  |  |  |
| All cause mortality | 7,571 (17.0%) | 7,241 (16.6%) | 7,128 (15.9%) | 6,534 (16.1%) |
| CV mortality | 6,040 (13.6%) | 5,720 (13.1%) | 5,662 (12.6%) | 5,183 (12.8%) |
| Any AMI hospitalisation | 2,710 (6.1%) | 2,559 (5.9%) | 2,394 (5.3%) | 2,076 (5.1%) |
| Any stroke hospitalisation | 1,656 (3.7%) | 1,580 (3.6%) | 1,620 (3.6%) | 1,510 (3.7%) |
| Any ACS hospitalisation | 4,432 (10.0%) | 4,187 (9.6%) | 3,854 (8.6%) | 3,321 (8.2%) |
| Any heart failure hospitalisation (HFH) | 2,951 (6.6%) | 2,879 (6.6%) | 2,857 (6.4%) | 2,634 (6.5%) |
| Any CV hospitalisation | 18,130 (40.8%) | 16,843 (38.6%) | 16,777 (37.5%) | 14,755 (36.3%) |
| MACE | 21,989 (49.5%) | 20,651 (47.3%) | 20,493 (45.8%) | 18,188 (44.8%) |

Supplementary Table 5: Instrumental Variable Coefficients (IVA) and Average Treatment Effect (ATE) with 95% Confidence Intervals by Outcome

| Outcome | Method | In-hospital | 1 Year | 3 Years | 5 Years |
| --- | --- | --- | --- | --- | --- |
| In-hospital mortality | IVA | 0.274 (0.141, 0.408) |  |  |  |
| In-hospital mortality | ATE | 0.011 (0.006, 0.016) |  |  |  |
| All-cause mortality | IVA |  | -0.015 (-0.112, 0.081) | -0.16 (-0.242, -0.078) | -0.245 (-0.319, -0.17) |
| All-cause mortality | ATE |  | -0.001 (-0.011, 0.008) | -0.026 (-0.04, -0.012) | -0.054 (-0.07, -0.037) |
| CV mortality | IVA |  | -0.152 (-0.221, -0.084) | -0.15 (-0.236, -0.064) | -0.229 (-0.307, -0.15) |
| CV mortality | ATE |  | -0.039 (-0.057, -0.021) | -0.021 (-0.034, -0.009) | -0.043 (-0.058, -0.028) |
| Any stroke hospitalisation | IVA |  | -0.34 (-0.534, -0.146) | -0.238 (-0.377, -0.099) | -0.18 (-0.297, -0.063) |
| Any stroke hospitalisation | ATE |  | -0.009 (-0.015, -0.002) | -0.013 (-0.021, -0.005) | -0.014 (-0.024, -0.004) |
| Acute myocardial infarction hospitalisation | IVA |  | -0.351 (-0.482, -0.22) | -0.482 (-0.59, -0.374) | -0.494 (-0.59, -0.397) |
| Acute myocardial infarction hospitalisation | ATE |  | -0.017 (-0.024, -0.01) | -0.04 (-0.05, -0.03) | -0.056 (-0.068, -0.044) |
| Acute coronary syndrome hospitalisation | IVA |  | -0.429 (-0.536, -0.322) | -0.577 (-0.667, -0.486) | -0.596 (-0.679, -0.512) |
| Acute coronary syndrome hospitalisation | ATE |  | -0.036 (-0.046, -0.026) | -0.077 (-0.091, -0.063) | -0.098 (-0.114, -0.083) |
| Heart failure hospitalisation | IVA |  | -0.039 (-0.168, 0.089) | -0.124 (-0.234, -0.013) | -0.165 (-0.266, -0.064) |
| Heart failure hospitalisation | ATE |  | -0.002 (-0.01, 0.005) | -0.011 (-0.021, -0.001) | -0.019 (-0.03, -0.007) |
| Repeat revascularisation >90days | IVA |  | -1.17 (-1.31, -1.03) | -1.19 (-1.36, -1.02) | - 1.12 (-1.28, -0.96) |
| Repeat revascularisation >90days | ATE |  | -0.094 (-0.11, -0.072) | -0.151 (-0.188, -0.114) | -0.168 (-0.206, -0.131) |
| Any CV hospitalisation | IVA |  | -0.69 (-0.751, -0.629) | -0.776 (-0.834, -0.719) | -0.761 (-0.818, -0.705) |
| Any CV hospitalisation | ATE |  | -0.201 (-0.221, -0.182) | -0.268 (-0.288, -0.247) | -0.275 (-0.295, -0.255) |
| MACE | IVA |  | -0.625 (-0.685, -0.566) | -0.752 (-0.809, -0.695) | -0.767 (-0.823, -0.711) |
| MACE | ATE |  | -0.196 (-0.216, -0.177) | 0.187 (0.149, 0.224) | -0.276 (-0.296, -0.257) |

Supplementary Table 6: Adjusted Hazard Ratios (aHR) and 95% Confidence Intervals by CABG:PCI Quartiles and Outcomes

| Outcome | CABG/PCI Quartile | 1 Year | 3 Years | 5 Years |
| --- | --- | --- | --- | --- |
| All cause mortality | Intermediate | 1.08 (1.02-1.14) | 1.01 (0.97-1.05) | 1 (0.97-1.03) |
| CV mortality | Intermediate | 0.98 (0.95-1) | 0.99 (0.95-1.04) | 0.99 (0.95-1.02) |
| AMI hospitalisation | Intermediate | 1.01 (0.92-1.11) | 0.97 (0.91-1.03) | 0.95 (0.9-1.01) |
| ACS hospitalisation | Intermediate | 0.97 (0.91-1.04) | 0.94 (0.89-0.99) | 0.94 (0.9-0.98) |
| HF hospitalisation | Intermediate | 0.98 (0.9-1.05) | 0.95 (0.89-1.01) | 0.94 (0.89-0.99) |
| CVA hospitalisation | Intermediate | 0.94 (0.82-1.09) | 0.96 (0.88-1.05) | 0.94 (0.87-1) |
| Any cardiovascular hospitalisation | Intermediate | 0.94 (0.91-0.96) | 0.92 (0.9-0.94) | 0.92 (0.9-0.94) |
| Major adverse cardiovascular events | Intermediate | 0.96 (0.93-0.98) | 0.94 (0.92-0.96) | 0.94 (0.92-0.96) |
| All cause mortality | High | 0.94 (0.89-1) | 0.93 (0.89-0.97) | 0.94 (0.91-0.97) |
| CV mortality | High | 0.97 (0.95-1) | 0.92 (0.88-0.96) | 0.93 (0.89-0.96) |
| AMI hospitalisation | High | 0.94 (0.85-1.03) | 0.91 (0.85-0.98) | 0.9 (0.85-0.96) |
| ACS hospitalisation | High | 0.87 (0.82-0.94) | 0.86 (0.82-0.91) | 0.88 (0.84-0.92) |
| HF hospitalisation | High | 0.95 (0.88-1.03) | 0.93 (0.87-0.99) | 0.93 (0.88-0.99) |
| CVA hospitalisation | High | 0.86 (0.74-1) | 0.95 (0.87-1.05) | 0.93 (0.86-1) |
| Any cardiovascular hospitalisation | High | 0.89 (0.86-0.92) | 0.89 (0.87-0.92) | 0.9 (0.88-0.92) |
| Major adverse cardiovascular events | High | 0.89 (0.87-0.92) | 0.89 (0.87-0.91) | 0.9 (0.89-0.92) |
| All cause mortality | Very High | 0.98 (0.92-1.04) | 0.93 (0.89-0.97) | 0.93 (0.9-0.96) |
| CV mortality | Very High | 0.97 (0.95-0.99) | 0.93 (0.88-0.97) | 0.92 (0.89-0.96) |
| AMI hospitalisation | Very High | 0.84 (0.76-0.93) | 0.84 (0.78-0.9) | 0.84 (0.79-0.89) |
| ACS hospitalisation | Very High | 0.82 (0.76-0.88) | 0.81 (0.77-0.85) | 0.81 (0.78-0.85) |
| HF hospitalisation | Very High | 1 (0.92-1.08) | 0.98 (0.92-1.04) | 0.97 (0.92-1.03) |
| CVA hospitalisation | Very High | 0.83 (0.71-0.96) | 0.94 (0.86-1.03) | 0.96 (0.9-1.03) |
| Any cardiovascular hospitalisation | Very High | 0.83 (0.8-0.85) | 0.83 (0.81-0.85) | 0.85 (0.83-0.87) |
| Major adverse cardiovascular events | Very High | 0.84 (0.82-0.87) | 0.84 (0.82-0.86) | 0.86 (0.84-0.87) |

Supplementary Table 7: Sensitivity Analyses Using Propensity Score Matching: Adjusted Coefficients of Long-term Outcomes Stratified by CABG:PCI Quartiles

| Outcome | CABG/PCI Quartile | 1 Year | 3 Years | 5 Years |
| --- | --- | --- | --- | --- |
| All cause mortality | Intermediate | 0.003 (0-0.007) | -0.003 (-0.007-0.001) | -0.002 (-0.005-0.001) |
| CV mortality | Intermediate | 0.001 (-0.001-0.003) | 0.002 (-0.003-0.007) | -0.004 (-0.009-0) |
| Any stroke hospitalisation | Intermediate | -0.012 (-0.018--0.006) | -0.003 (-0.006-0) | 0.002 (-0.004-0.007) |
| Heart failure hospitalisation (HFH) | Intermediate | 0.003 (-0.001-0.006) | -0.022 (-0.03--0.015) | -0.004 (-0.008--0.001) |
| Acute myocardial infarction (AMI) | Intermediate | 0 (-0.003-0.003) | 0 (-0.004-0.004) | -0.025 (-0.032--0.017) |
| Any acute coronary syndrome (ACS) | Intermediate | -0.01 (-0.016--0.003) | 0 (-0.003-0.003) | 0.001 (-0.006-0.004) |
| Any CV hospitalisation (CVH) | Intermediate | 0 (-0.002-0.001) | -0.02 (-0.028--0.013) | -0.001 (-0.004-0.003) |
| Major adverse cardiovascular events (MACE) | Intermediate | -0.001 (-0.003-0.002) | -0.002 (-0.004-0.001) | -0.02 (-0.028--0.013) |
| All cause mortality | High | -0.006 (-0.009--0.003) | -0.009 (-0.013--0.005) | -0.001 (-0.004-0.002) |
| CV mortality | High | -0.001 (-0.003-0.001) | -0.011 (-0.015--0.006) | -0.01 (-0.015--0.006) |
| Any stroke hospitalisation | High | -0.024 (-0.031--0.018) | -0.002 (-0.006-0.001) | -0.012 (-0.018--0.007) |
| Heart failure hospitalisation (HFH) | High | -0.006 (-0.009--0.002) | -0.032 (-0.039--0.025) | -0.003 (-0.006-0.001) |
| Acute myocardial infarction (AMI) | High | -0.004 (-0.007--0.001) | -0.01 (-0.014--0.005) | -0.033 (-0.04--0.026) |
| Any acute coronary syndrome (ACS) | High | -0.028 (-0.035--0.022) | -0.003 (-0.006--0.001) | -0.012 (-0.017--0.007) |
| Any CV hospitalisation (CVH) | High | -0.001 (-0.003-0) | -0.039 (-0.046--0.031) | -0.005 (-0.008--0.001) |
| Major adverse cardiovascular events (MACE) | High | -0.001 (-0.003-0.002) | -0.001 (-0.003-0.002) | -0.038 (-0.046--0.031) |
| All cause mortality | Very High | -0.004 (-0.008--0.001) | -0.013 (-0.017--0.009) | -0.001 (-0.004-0.002) |
| CV mortality | Very High | -0.003 (-0.005--0.001) | -0.012 (-0.016--0.007) | -0.017 (-0.022--0.013) |
| Any stroke hospitalisation | Very High | -0.037 (-0.043--0.03) | -0.002 (-0.006-0.001) | -0.015 (-0.02--0.009) |
| Heart failure hospitalisation (HFH) | Very High | -0.004 (-0.007-0) | -0.048 (-0.055--0.04) | -0.002 (-0.005-0.002) |
| Acute myocardial infarction (AMI) | Very High | -0.007 (-0.011--0.004) | -0.012 (-0.016--0.007) | -0.048 (-0.056--0.041) |
| Any acute coronary syndrome (ACS) | Very High | -0.041 (-0.048--0.034) | -0.005 (-0.008--0.002) | -0.015 (-0.02--0.01) |
| Any CV hospitalisation (CVH) | Very High | -0.001 (-0.003-0) | -0.054 (-0.061--0.046) | -0.008 (-0.012--0.005) |
| Major adverse cardiovascular events (MACE) | Very High | -0.001 (-0.004-0.002) | -0.002 (-0.004-0.001) | -0.053 (-0.061--0.046) |

**Supplementary Figure 1:** STROBE Diagram Depicting the Inclusion and Exclusion of Patients with Multivessel Coronary Artery Disease Undergoing Revascularization, Along with Follow-up Completion at 1 and 5 Years


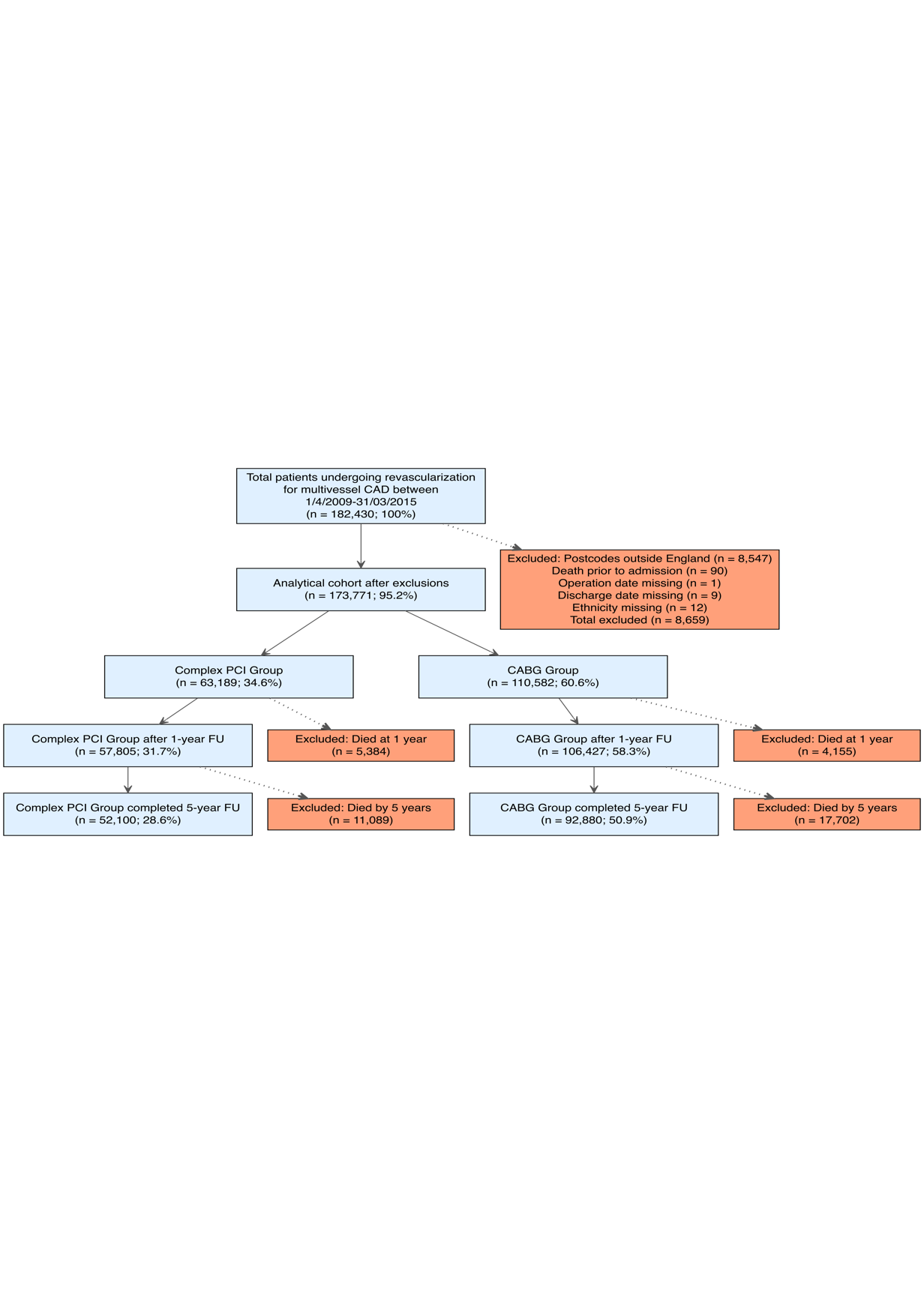


**Supplementary Figure 2:** Heat map reporting regional CABG: PCI rates aggregated by clinical commission groups

**
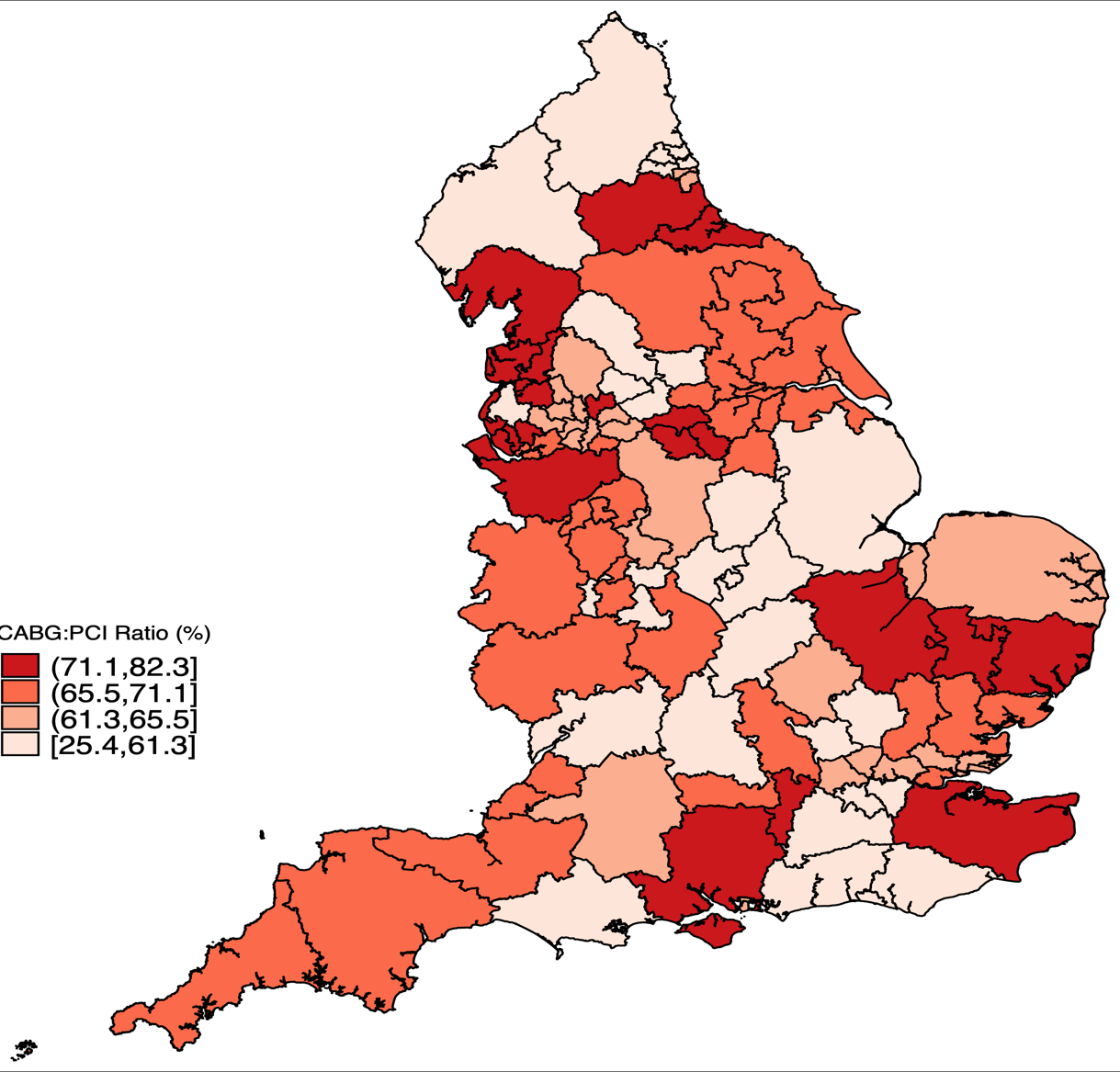
**

CABG=coronary artery bypass graft, PCI=percutaneous coronary intervention.

**Supplementary Figure 3:** Temporal Trends in proportion of CABG in multivessel cohort across patient postcode areas.


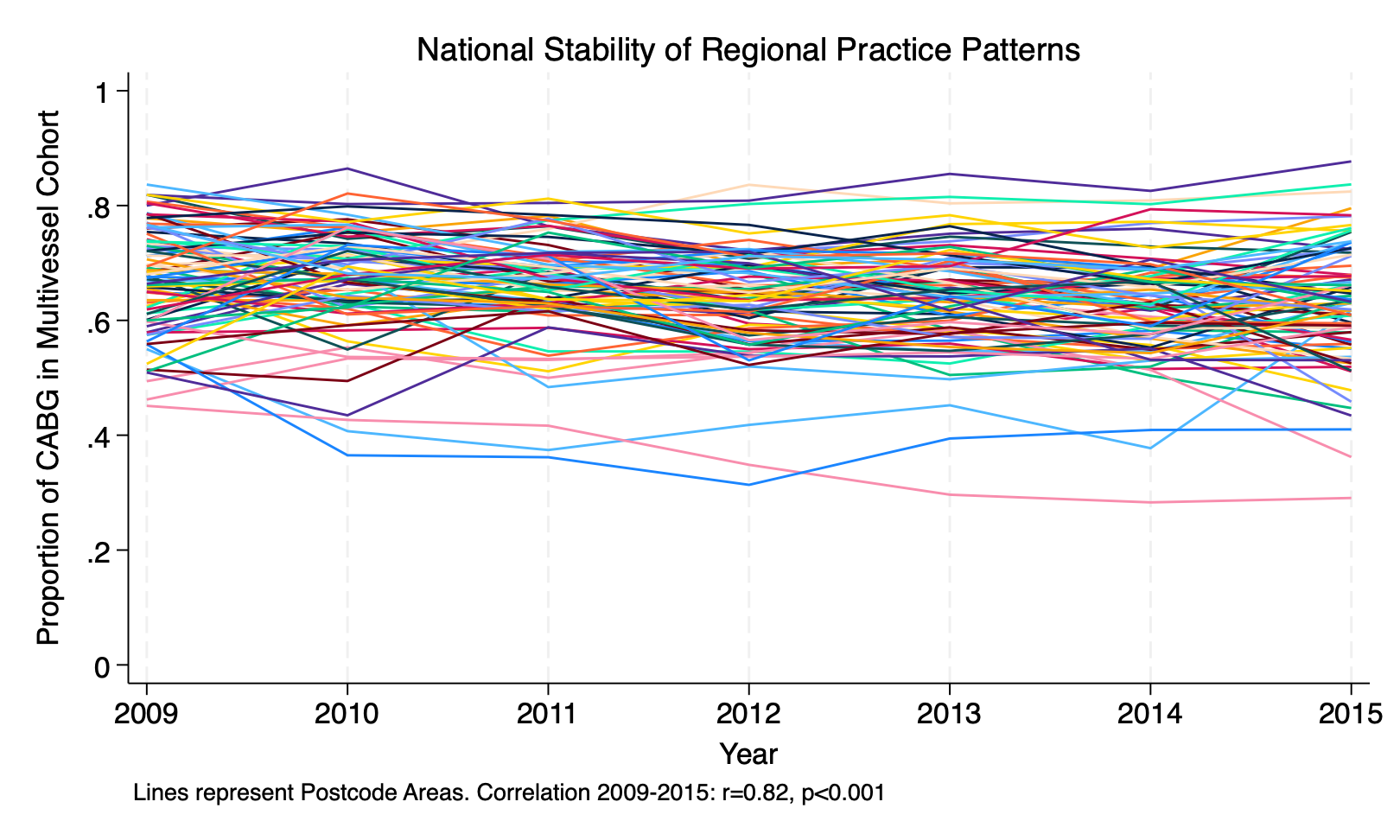


**Supplementary Figure 4:** Adjusted Hazard Ratios for five year outcomes, Comparing Multivessel PCI and CABG


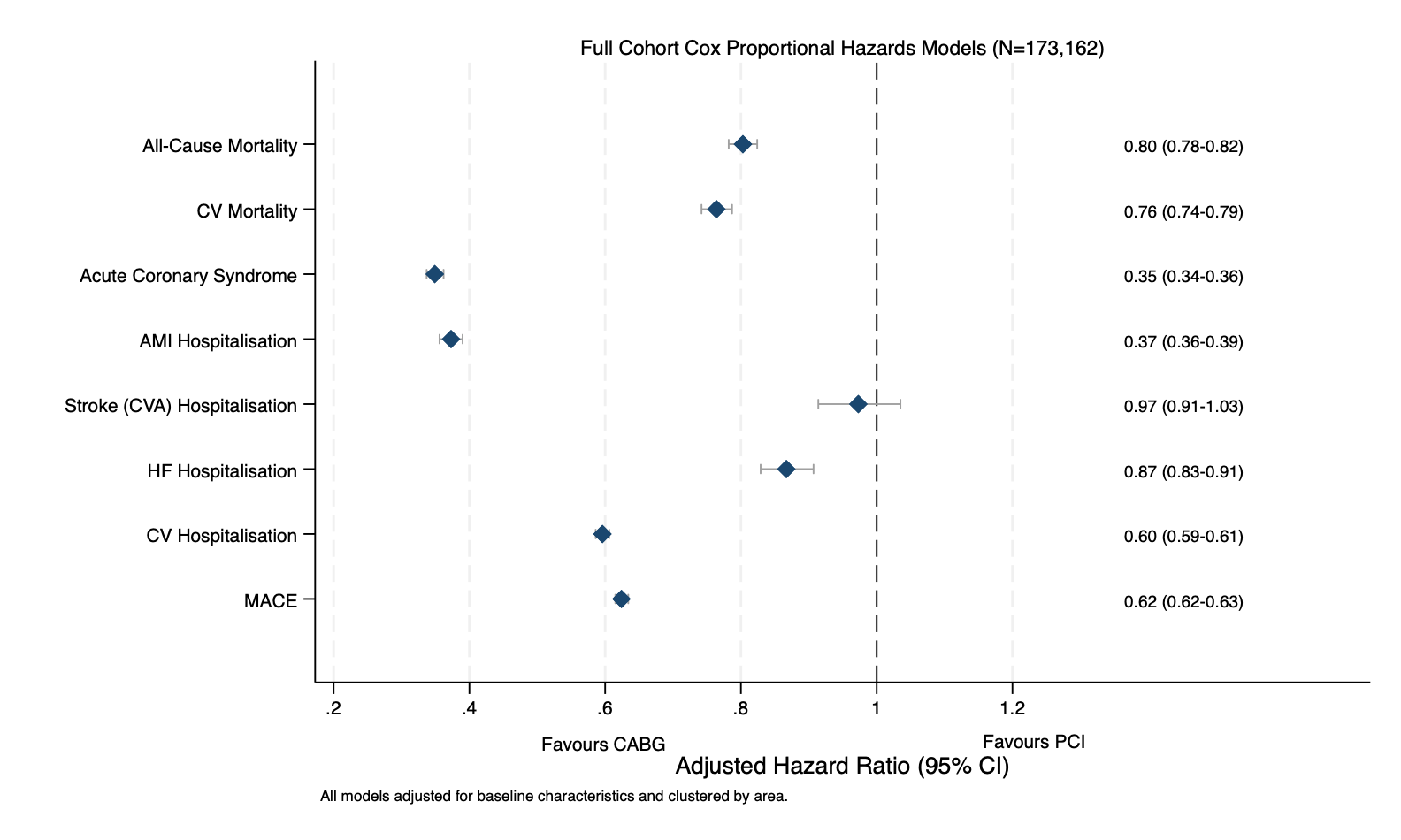


**Supplementary Figure 5:** Adjusted Hazard Ratios for All-Cause Mortality Across Subgroups Comparing Multivessel PCI and CABG


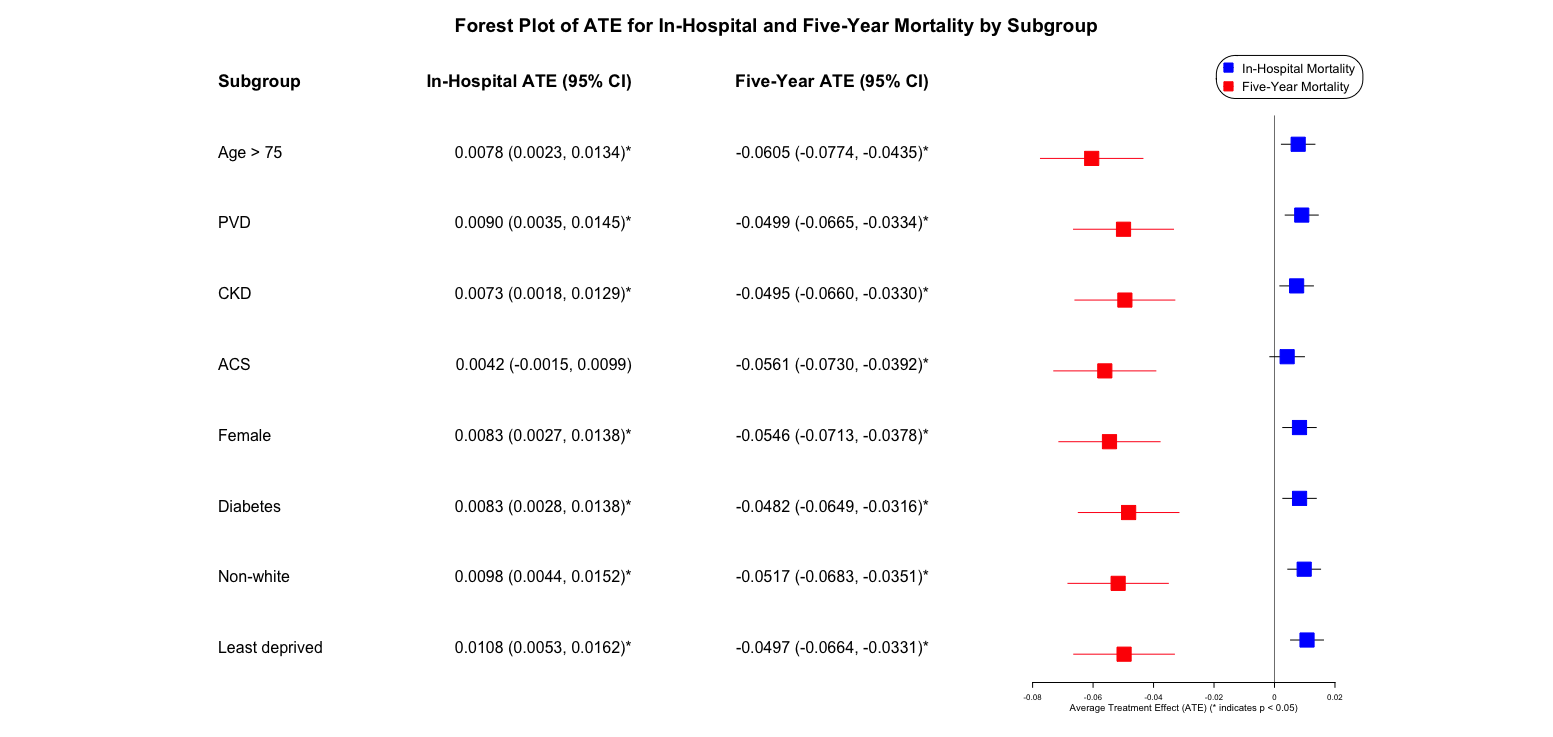


CABG=coronary artery bypass graft, PCI= percutaneous coronary intervention, ATE= average treatment effect, CKD= chronic kidney disease, PVD= peripheral vascular disease, ACS= acute coronary syndrome,

**Supplementary Figure 6:** Regional Variation in Predicted 5-Year Mortality Reduction: CABG vs. Multivessel PCI


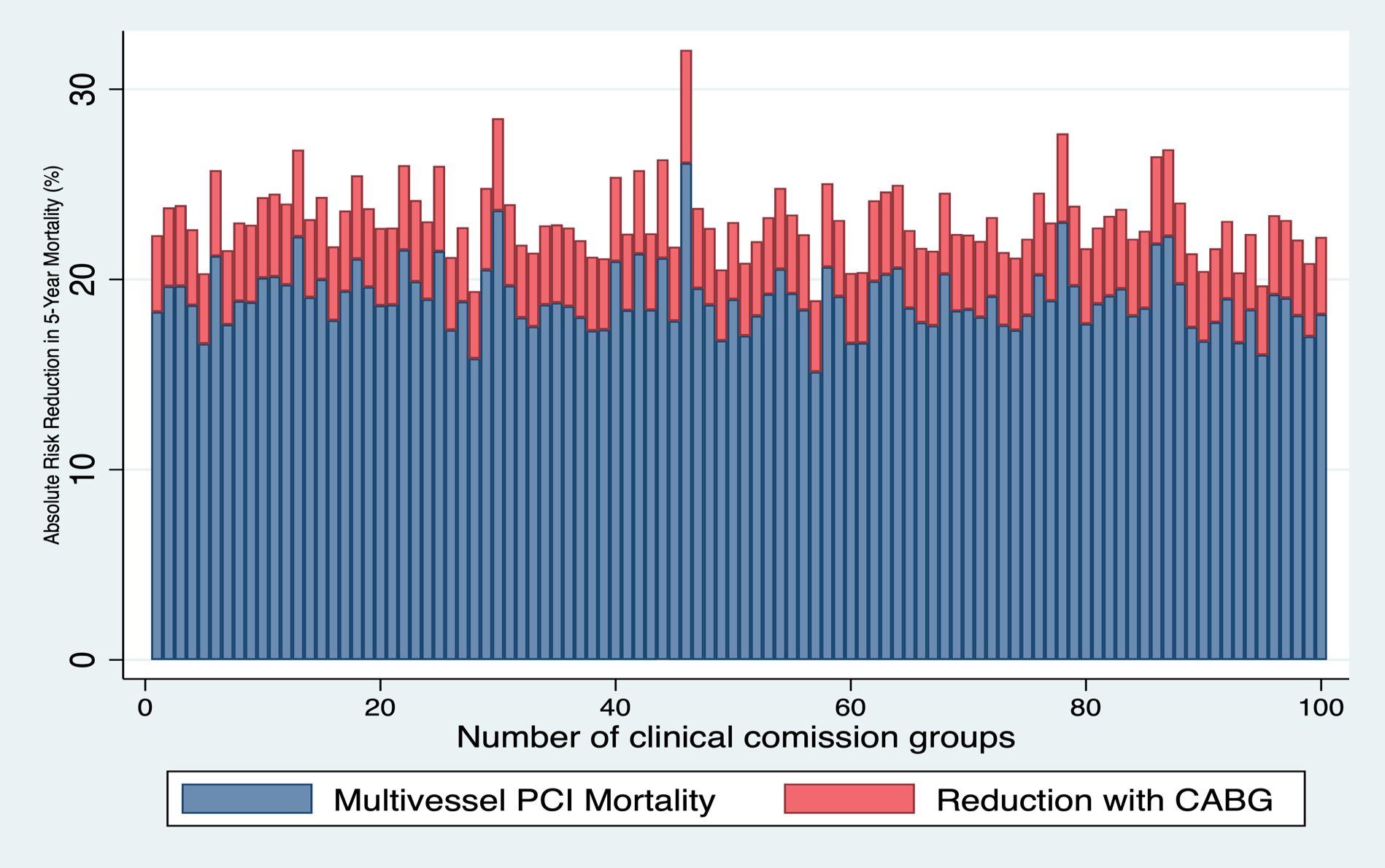

Supplement: oeag043_Supplementary_Data [file oeag043_supplementary_data.docx]
